# Women’s Satisfaction with Midwifery-Led Continuity of Care: Insights from Antenatal, Intrapartum, and Postnatal Experiences in the Sidama Region, Ethiopia. A Non-Randomized Control Trial

**DOI:** 10.1177/11786329251404113

**Published:** 2026-02-24

**Authors:** Rekiku Fikre, Sanne Gerards, Wondwosen Teklesilasie, Jessica Gubbels

**Affiliations:** 1 Department of Health Promotion, Faculty of Health, Medicine and Life Sciences, NUTRIM Institute of Nutrition and Translational Research in Metabolism, Maastricht University, The Netherlands; 2 Department of Midwifery, College of Medicine and Health Sciences, Hawassa University, Ethiopia; 3 School of Public Health, College of Medicine and Health Sciences, Hawassa University, Ethiopia

**Keywords:** women’s, satisfaction, midwifery-led continuity of care, Ethiopia

## Abstract

**Background:**

Evidence from high-income countries indicates that midwifery-led continuity of care improves maternal satisfaction and health outcomes throughout pregnancy and postpartum. In Ethiopia, low maternal satisfaction with care indicates systemic problems, including ineffective communication and substandard service quality. This study aimed to evaluate how midwifery-led continuity care impacts maternal satisfaction during antenatal, intrapartum, and postpartum care in the Sidama region’s general hospitals in Ethiopia.

**Methods:**

A prospective non-randomized controlled trial was conducted from October 2023 to June 2024 in four general hospitals in the Sidama region of Ethiopia. A total of 478 low-risk women were allocated into two groups: midwifery-led continuity of care (n = 239 and shared care n = 239). Quantitative data were gathered through face-to-face interviews to evaluate maternal satisfaction, and average satisfaction scores between groups were analyzed using bootstrapping linear regression.

**Results:**

Women in the midwifery-led continuity of care reported significantly higher satisfaction with continuity of care compared to shared care, with an adjusted mean difference of 0.28 (95% CI 0.13-0.40; *P* < .001). Specifically, during antenatal care, the adjusted mean difference was 0.20 (95% CI 0.06-0.33; *P* < .003), intrapartum care was 0.31 (95% CI 0.17-0.45; *P* < .001), and postnatal care was 0.44 (95% CI 0.29-0.59; *P* < .001).

**Conclusion:**

Women receiving midwifery-led continuity of care reported higher satisfaction levels, making this model a viable alternative for improving satisfaction in maternity services by offering a more personalized and supportive approach to care.

## Introduction

Maternal and newborn health remains a crucial global health priority, marked by significant regional disparities in healthcare access and quality between developing and developed countries.^1^ Globally, the maternal mortality ratio has been significantly reduced from 953 per 100 000 live births in 2000 to 267 in 2020.^2,3^ Despite this progress, approximately 800 women still die daily from preventable causes related to pregnancy and childbirth, with 75% of these deaths occurring in sub-Saharan Africa.^2^ In Ethiopia, the maternal mortality rate decreased from 871 deaths per 100 000 live births in 2000 to 267 in 2020, with more recent figures indicating continued improvement, estimating the rate at approximately 195 per 100 000 live births in 2023.^4^

The Sustainable Development Goals (SDGs), adopted by the United Nations in 2015, are a global call to end poverty, protect the planet, and ensure peace and prosperity by 2030.^5^ SDG 3 focuses on ensuring healthy lives, with specific targets to reduce maternal mortality to less than 70 deaths per 100 000 live births and to end preventable newborn and child deaths, aiming to cut neonatal mortality to 12 per 1000 live births.^5^ Despite progress in reducing maternal and newborn mortality rates, the current pace is insufficient to fully achieve the SDG targets by 2030, highlighting ongoing challenges in healthcare access and quality worldwide.^5^

Maternal satisfaction is a key indicator of healthcare quality, reflecting how well care aligns with women’s expectations regarding care during pregnancy, childbirth, and postpartum.^6^ High satisfaction levels correlate positively with perceived care quality, leading to better follow-up attendance and health practices, which in turn lead to improved overall maternal and neonatal health outcomes.^7,8^ Evidence shows that higher levels of satisfaction are associated with lower rates of cesarean sections and episiotomies, improved maternal psychological well-being, and women feeling more empowerment and autonomy during childbirth.^7,9-11^ However, in low- and middle-income countries, less than 50% of women report high satisfaction with maternity services.^12^ A low satisfaction level adversely affects service utilization and overall care quality, consequently leading to delays in seeking care for complications and decreased utilization of essential services like antenatal care and facility deliveries.^13^ Recent research in Ethiopia indicates that maternal satisfaction with maternity care services is low, which is linked to systemic problems like limited resources, poor service quality, and lack of privacy and respect.^12^ This dissatisfaction underscores the urgent need for comprehensive improvements in healthcare and innovative approaches to address these gaps effectively.

Midwifery-led continuity of care is a model where midwives take primary responsibility for providing continuous, comprehensive care to women throughout pregnancy, childbirth, and the postnatal period.^1^ While shared care refers to a collaborative healthcare arrangement where midwives, nurses, obstetricians, and other health professionals jointly manage a woman’s maternity care.^1^

Midwife-led continuity of care (MLCC) enhances maternal satisfaction by fostering personalized support, building a trusting relationship that enhances women’s confidence and emotional well-being during pregnancy, childbirth, and postpartum, and reducing interventions throughout pregnancy, childbirth, and postpartum.^14^ Studies show that MLCC leads to higher satisfaction scores compared to shared care models, as women feel more supported and understood during their care journey.^15-18^

Most research on maternal satisfaction with MLCC has been conducted in high-income countries,^14,19,20^ leaving a gap in knowledge about low-resource settings, where maternal and newborn mortality rates are high, and service quality is often low.^7^ The MLCC model is likely a viable strategy for enhancing maternal satisfaction in low-resource settings like Ethiopia.^16,21^ This study therefore aims to investigate and compare the satisfaction levels of women receiving midwife-led continuity of care (MLCC) versus those receiving shared care during antenatal, intrapartum, and post-partum care received in general hospitals of the Sidama region in Ethiopia.

## Methods

### Study Design and Setting

A prospective non-randomized controlled trial design was conducted from October 2023 to June 2024 in four general hospitals of the Sidama region, Ethiopia. The study investigates and compares the satisfaction levels of women receiving midwife-led continuity of care (MLCC) versus those receiving shared care during antenatal, intrapartum, and post-partum care received in general hospitals of the Sidama region in Ethiopia.

The Sidama region is organized into thirty-six districts, with Hawassa city serving as the city administration. Sidama is bordered by Oromia to the north, east, and south, and by Wolayita to the west. Sidama has an estimated total population of 4 469 455.^22^ Of the 21 hospitals in the Sidama region, four were randomly selected for the current study. Each hospital has an average of 20 to 25 midwives.

### Eligibility Criteria

Women who were less than 24 weeks pregnant during their initial antenatal care (ANC) visit and had a low obstetric risk (single pregnancy, term pregnancy, no previous maternal complications, absence of pre-existing medical and obstetric complications) were included for the study. Those with a history of medical or obstetric complications based on the national ANC guideline,^23^ as well as those planning to schedule with another care provider other than midwives (eg, nurses, health officers, emergency surgeons, general practitioners, and obstetricians), were excluded from the current study.

### Sample Size Determination

The required sample size was determined using Open-Epi version 7.2.5, based on the assumptions of a 95% confidence interval (CI), 80% power, and a ratio of exposed to unexposed participants to one. To calculate the appropriate sample size, a study conducted in another region in Ethiopia (North Shoa) on the effects of MLCC on maternal and neonatal health outcomes was used.^21^ This particular study found that the percentage of preterm births in the intervention group was 6.4%, while the control group had a rate of 15.2%.^16^ With a two-sided confidence level of 95% (CI) and a power of 80%, the ratio of mothers receiving MLCC to those receiving shared care was calculated. Assuming a 10% loss to follow-up, the total required sample size was determined to be 478 pregnant women, with 239 assigned to the 2 hospitals in the control group (shared care) and 239 to the 2 intervention hospitals (MLCC).

### Selection Procedure and Sampling Techniques

Four general hospitals were randomly selected for the study, and the calculated sample size was proportionally distributed among these hospitals. The hospitals included in this study have a comparable governance structure and provide the same range of services.^22^ To estimate the number of pregnant women seeking services, we analyzed antenatal care data from 3 months from each hospital before the beginning of the intervention. Participants were selected using a systematic random sampling technique with an interval of two. The first participant in each hospital was chosen by a lottery method. The hospitals chosen for the intervention were selected due to having a sufficient number of volunteer midwives available to implement the MLCC model, and the remaining hospitals were control hospitals. Women who met the inclusion criteria and expressed a willingness to participate were selected until the desired sample size was reached in both groups. During the study period, a total of 1319 mothers visited antenatal care clinics.

**Figure 1.**
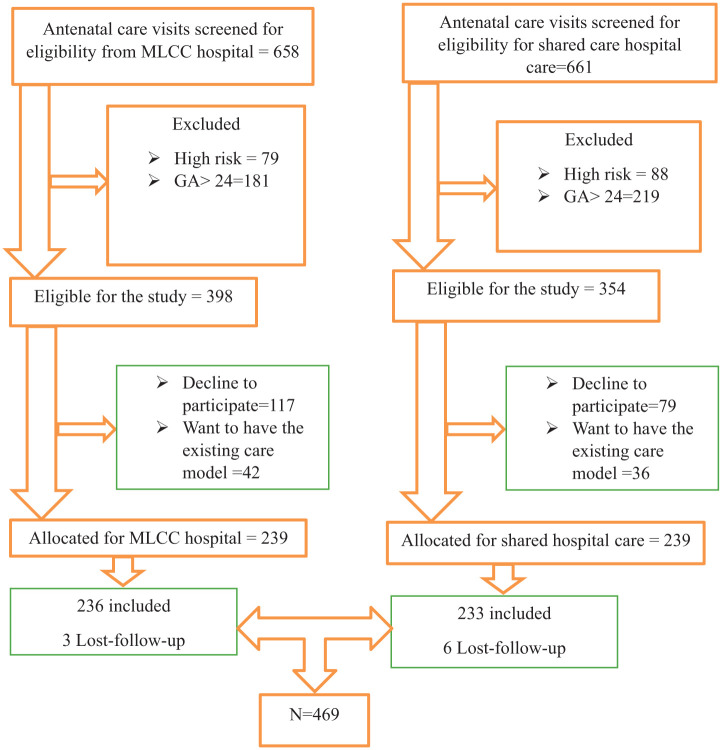
Study participant’s recruitment process from October 2023 to June 2024, in the Sidama Region, Ethiopia 2024. Abbreviations: GA, gestational age in weeks; MLCC, midwifery-led continuity of care.

Out of these, 658 women were found to be eligible for inclusion in the interventional study, and the remaining 661 participated shard care. However, from 658 eligible women in the interventional group, 115 women chose not to participate, and 31 preferred to give birth with other health professionals. In total, 478 women (76.6%) participated in the study, with 239 assigned to the MLCC group and 239 to the shared model of care group. In the data analysis, 236 women remained in the MLCC group, as three were lost to follow-up, and 233 remained in the shared care group, with six lost to follow-up (see Figure 1).

### Intervention

#### The Intervention

Women involved in the study at the intervention hospitals received MLCC from participating midwives during their pregnancy, labor, delivery, and immediate postpartum period. These midwives received specialized training for 1 week, focusing on an approach that prioritizes continuity of care and equipping them to support women throughout their maternity journey in accordance with the MLCC model.^14^ In this group, the midwives were responsible for ensuring continuous care for each woman and supported one another in the event of a primary midwife’s absence. In MLCC, if complications arise, the primary midwife works in collaboration with other healthcare professionals to ensure continued support.^14^ One midwife from each MLCC team was assigned to the labor ward to offer care during childbirth. In the case of women undergoing cesarean sections, midwives extended their care to the operating theater. Following the delivery, the MLCC model continued into the postnatal ward. Each day, one midwife from every team was designated to the postnatal ward, ensuring continuity of care for women by a midwife from their own care team during their hospital stay after giving birth.

#### Control Group

Pregnant women who received antenatal, labor, birth, and postnatal care according to the established practice in Ethiopia. Women from the control hospitals received care using standard care practices for antenatal, labor, birth, and postnatal care (shared care). In this group, care was provided according to the shared model of care, which involves a distribution of responsibilities among various obstetric care providers.^24^ In this model, responsibility for care is distributed among various healthcare providers, including midwives, nurses, health officers, and doctors. Care is delivered through conventional 8-hour shifts, with healthcare providers handing over patients to the next shift’s staff. After discharge, each woman in this group received care from a different team of midwives or nurses who were assigned to family planning and immunization unit. This approach results in fragmented care, with multiple providers involved at different stages, limiting continuity and personalized support throughout the maternity period. The SMC model contrasts with midwifery-led continuity care, where a single provider or small team offers continuous support from pregnancy through postpartum.

### Outcome Variables

Maternal satisfaction with antenatal, intrapartum, and postnatal care was used as the outcome variable. This was evaluated using a total of 24 questions to determine maternal satisfaction throughout the continuity of care. Maternal satisfaction was operationalized as women’s relationships with midwives, their involvement in decision-making, and their perceptions of care quality. Responses were recorded on a five-point Likert scale, ranging from 1 (strongly disagree) to 5 (strongly agree). The responses for antenatal, intrapartum, and early postnatal care were summarized, and the mean satisfaction scores for both groups were compared. Several socio-demographic factors were assessed as predictor variables: mother’s age in years, age at first marriage (in years), educational status of the women (no formal education, primary education, secondary education, and college level and above), residency of the mother (rural or urban), occupation of the mother (student, housewife, or employed), as well as past obstetric and gynecological history (encompassing parity, gravidity, age at first pregnancy (in years), history of contraceptive use (yes/no), planned pregnancy (yes/no), and wanted pregnancy (yes/no).

### Data Collection

The data collection tool for this study was adapted from the satisfaction with antenatal, intrapartum, and postpartum care tools.^21^ The data collection tools were initially prepared in English, then translated into the local language (Sidama Afoo) by a language expert. The translated version was then back-translated into English by two other independent translators unaware of the original questionnaire to check for accuracy. Finally, the translated questionnaire was pilot tested with a sample of the target population to verify that the items were clearly understood and retained the meaning of the original questionnaire. The internal consistency of the data collection instrument was assessed using Cronbach’s alpha. The satisfaction scores were as follows: antenatal care (Cronbach alpha = .89), intrapartum care (Cronbach alpha = .88), and postnatal care (Cronbach alpha = .94). The baseline characteristics of the study participants, including sociodemographic information, and previous obstetric and gynecologic history, as well as medical and surgical details, were systematically documented by midwives at each hospital using a standardized data collection tool designed for this study. Six midwives were recruited as data collectors, and two supervisors with master’s degrees in clinical midwifery were appointed.

The data collectors received 2 days of training to familiarize them with the tools and ensure they clearly understood the study’s objectives. Structured face-to-face interviews were conducted at the end of the postnatal period using standardized, interviewer-administered questionnaires. All details of the women were de-identified to ensure that individuals could not be identified.

## Data Analysis

The dataset was obtained from the Kobo Toolbox server and then exported to Microsoft Excel 2013. Subsequently, it was imported into Stata version 16 for data cleaning, recording, and analysis. Summary statistics were used to describe the socio-demographic, and obstetric characteristics as well as the preferences of the study participants. Various statistical methods were employed to analyze differences in characteristics between the intervention and control groups. Independent samples *t*-tests were used to compare group means and determine whether statistically significant differences existed. When the assumptions for parametric tests were not met, Mann-Whitney *U* tests were applied to assess differences in the distributions between the two groups. In addition, Fisher’s exact tests were used to evaluate associations between categorical variables. The average satisfaction sum-scores between the groups were analyzed using bootstrapping linear regression. The main outcome measured was the mean satisfaction sum score throughout antenatal, intrapartum, or postnatal care. Factors that might affect the differences between the groups were included for adjustment in tables four, five, and six. Adjusted bias-corrected and accelerated bootstrap estimates with 95% confidence intervals were provided for non-normally distributed ordinal outcomes, utilizing 10 000 bootstrap samples. A significant level of .05 was established.

### Ethical Clearance

Ethical approvals were secured from the Institutional Review Board of Hawassa University College of Medicine and Health Sciences (Ref. no. IRB/080/14), and authorization was granted by the Sidama Regional Public Health Institute. A written informed consent was obtained from each participant before data collection began, and also a written informed consent was obtained from the parents or legal guardians of all participants under 18 years of age, and all procedures were conducted in accordance with the *Declaration of Helsinki*.^25^ This consent process involved providing clear, understandable information about the study’s objectives, procedures, risks, and benefits, and the consent was documented by signed consent forms retained securely by the research team.

This consent was acquired after providing a detailed explanation of the study’s aims, data collection methods, potential benefits and risks of participation, and the voluntary nature of their involvement in the research.

## Results

### Socio-Demographic Characteristics

There were various significant differences in the demographic characteristics between the intervention group (MLCC) and the control group in all socio-demographic characteristics assessed (see Table 1). In the MLCC group, participants were more often in the age category of 18 to 25 years, more often lived in urban areas, more often had completed secondary education, and more often were housewives, compared to the shared care group. Regarding the age at first marriage, 77 women (32.6%) in the MLCC group got married between the ages of 16 and 18, which is significantly lower than the percentage observed in the shared care group (39.1%). Further, more participants in the MLCC group identified as Sidama ethnicity. The Sidama are one of the largest ethnic groups in the region, distinguished by their unique language and cultural practices. Women in MLCC group reported a monthly income exceeding 35.386 USD, compared to the shared care group.

**Table 1.** Sociodemographic Characteristics of Study Participants Among Intervention and Control Groups, Sidama Regional State, Ethiopia.

Variable	Category	Midwifery-led continuity of care (N = 236 (100%))	Shared care (N = 233 (100%))	*P*-value
Age (in years)	<18	57 (24.2)	54 (23.2)	.038
	18-25	95 (40.3)	70 (30.0)	
	⩾25	84 (35.6)	109 (46.8)	
	Mean (SD)	26.2 (5.2)	25.4 (4.8)	
Age (in years) at first marriage	16-18	77 (32.6)	91 (39.1)	.021
	19-21	98 (41.5)	77 (33)	
	22-24	38 (16.1)	55 (23.6)	
	⩾25	23 (9.7)	10 (4.3)	
	Mean (±SD)	20.1 (2.7)	19.9 (2.5)	
Residency	Rural	15 (6.4)	97 (41.6)	<.001
	Urban	221 (93.6)	136 (58.4)	
Educational status	No formal education	8 (3.4)	30 (12.8)	<.001
	Primary education	26 (11.0)	27 (11.6)	
	Secondary education	138 (58.5)	104 (44.6)	
	College level and above	64 (27.1)	72 (30.9)	
Occupation	Student	17 (7.2)	33 (14.2)	<.001
	Housewife	187 (79.2)	77 (33.0)	
	Employed	32 (12.9)	116 (49.8)	
	Others*^a^	0 (0)	7 (3.0)	
Ethnicity	Sidama	210 (89.0)	128 (54.9)	<.001
	Amhara	10 (4.2)	24 (10.3)	
	Other	16 (6.7)*^b^	80 (34.8)*^c^	
Religion	Protestant	200 (84.7)	124 (53.2)	<.001
	Ethiopian, Orthodox	21 (8.9)	66 (28.3)	
	Muslim	15 (6.3)	19 (8.2)	
	Catholic	0 (0)	22 (9.4)	
	Other*	0 (0)	2 (0.9)	
Monthly income in Ethiopian Birr	1000-3000	37 (15.7)	2 (0.9)	<.001
	3001-5000	108 (45.8)	24 (10.3)	
	⩾5001	91 (38.6)	207 (88.8)	

Shared care occupation other*^a^ (farmer and merchant), midwifery-led continuity of care ethnicity other*^b^ (Wolayita, Oromo, Gurage, and Hadiya), and shared care ethnicity other*^c^ (Gurage, Hadiya, Kambata and Tigre) shared care religion other * (only Jesus) *P*-value = *x*2 SD (standard deviation).

### Obstetrics and Gynecologic Characteristics

There were several significant differences in obstetric and gynecologic characteristics of the participants. In the MLCC group, 106 women (44.9%) had their first pregnancy between the ages of 19 and 21, while this figure was 112 women (48.1%) in the shared care group. The majority of women in both the MLCC group (88.1%) and the shared care group (80.3%) reported that their pregnancies were wanted (see Table 2).

**Table 2.** Participant’s Obstetrics and Gynecologic Characteristics, Sidama Regional State, Ethiopia.

Variable	Category	Midwifery-led continuity of care (N = 236 (100%))	Shared care (N = 233 (100%))	*P*-value
Pregnancy wanted	Yes	208 (88.1)	187 (80.3)	.001
	No	28 (11.8)	46 (19.7)	
Pregnancy planned	Yes	197 (83.5)	181 (77.7)	.037
	No	39 (16.5)	52 (22.3)	
Contraceptive use	Yes	157 (66.5)	187 (80.3)	.001
	No	79 (33.5)	46 (19.7)	
Abortion history	Yes	34 (14.4)	4 (1.7)	.001
	No	202 (85.6)	229 (98.3)	
Gravidity	Prim gravidity	97 (41.1)	114 (48.9)	.622
	Multigravida	139 (58.9)	119 (51.1)	
Age at first pregnancy	16-18	45 (19.1)	19 (8.2)	.032
	19-21	106 (44.9)	112 (48.1)	
	22-24	51 (21.6)	83 (35.6)	
	⩾25	34 (14.4)	19 (8.2)	
	Mean (±SD)	21.1 (2.8)	21.4 (2.3)	

### Women’s Perception About Continuity of Care

With regard to women’s experiences with the MLCC intervention, 219 women (92.8%) preferred to see the same midwives at each checkup. Almost all women receiving MLCC (98.7%) stated that having the same midwife throughout their antenatal care, labor, and postnatal periods was very important to them. Regarding continuity during labor, most women, 213 (90.3%) wanted to know the midwife who would care for them during labor, specifically the same midwife they had seen during their antenatal visits. Additionally, concerning postnatal care, 185 women (78.4%) expressed a strong preference to see the midwife who provided care during their intrapartum period during their postnatal visits (see Table 3).

**Table 3.** MLCC Participants’ Preference on Continuity of Care in Sidama Regional States, Ethiopia.

Variables	Categories	Midwifery-led continuity of care model (N = 236 (100%))
Do you prefer to see the same team of midwives at each checkup?	Yes	219 (92.8)
	No	17 (7.2)
Would you want to be cared for during birth by a team of midwives, which includes the midwives that you already met in antenatal care?	Strongly agree	213 (90.3)
	Agree	23 (9.7)
	Neutral	0 (0)
	Disagree	0 (0)
	Strongly disagree	0 (0)
Would you want to be cared postnatal care by team of midwives that you have already met in intrapartum?	Strongly agree	185 (78.4)
	Agree	51 (21.6)
	Neutral	0 (0)
	Disagree	0 (0)
	Strongly disagree	0 (0)
Is it important for you to have the same team of midwives throughout the pregnancy and postpartum period?	Not important	3 (1.3)
	Very important	233 (98.7)

### Satisfaction with All Care

**Figure 2.**
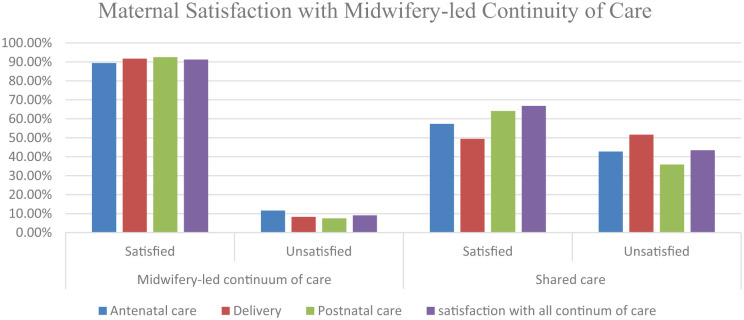
Maternal satisfaction with care in the MLCC (N = 236) and shared care group (N = 233).

In total, 89.4% of women within the MLCC group reported satisfaction with their antenatal care, in contrast to 57.3% of women in the shared care group (difference *P* = .001). In the MLCC group 91.7 % of women reported satisfaction with their intrapartum care, and 92.5% were satisfied with their postnatal care, in contrast to, 49.4% for intrapartum care (*P* = .001) and 64.1% for postnatal care (*P* = .001) in the shared care group, see Figure 2.

### Care During Pregnancy

The study found statistically significant differences in satisfaction with care between the MLCC and shared care groups receiving antenatal care. The MLCC group demonstrated a higher crude mean satisfaction score of 2.75, compared to a score of 2.54 in the shared care group throughout the entire course of pregnancy. Women in the MLCC group were, among others, more likely to report the following compared to those in the shared care: Midwives kept them informed about their pregnancy (adjusted mean difference 0.36, 95% CI 0.36 (0.18-0.54) ; *P* < .001), they had an active role in decision-making regarding their care during pregnancy (adjusted mean difference 0.28, 95% CI 0.28 (0.11-0.46); *P* < .001), and they received reassurance when needed (adjusted mean difference o.38, 95% CI 0.22-0.53; *P* < .001), see Table 4.

**Table 4.** Participant’s Satisfaction with Care During Pregnancy in Midwifery-Led Continuum of Care Compared to Shared Care.

Care assessed	Mean sum^a^ score midwifery-led continuity of care (N = 236 (100%))	Shared care (N = 233 (100%))	Crude mean difference (95% CI)	Adjusted mean^b^ difference (95% CI)	*P*-value
During my check-ups, I was consistently asked if I had any questions	2.65 (0.75)	2.47 (0.87)	0.18 (0.027-0.32)	0.30 (0.13-0.47)	<.001
The midwife/doctor consistently kept me updated and informed about the progress of my pregnancy and any relevant developments.	2.70 (0.71)	2.31 (0.94)	0.39 (0.23-0.54)	0.36 (0.18-0.54)	<.001
I was consistently involved in making decisions regarding my care during pregnancy.	2.74 (0.67)	2.35 (0.93)	0.39 (0.24-0.54)	0.28 (0.11-0.46)	<.001
The midwives/doctor provided reassurance when I needed it.	2.85 (0.52)	2.47 (0.88)	0.38 (0.25-0.51)	0.38 (0.22-0.53)	<.001
Care in pregnancy was provided in a safe and competent way.	2.61 (0.78)	2.42 (0.91)	0.19 (.044-0.35)	0.17 (0.036-0.32)	<.001
Overall care during pregnancy was very good.	2.88 (0.46)	2.55 (0.83)	0.32 (0.20-0.44)	0.32 (0.17-0.46)	<.001
Satisfaction with all care during pregnancy	2.75 (0.51)	2.54 (0.73)	0.21 (0.09-0.32)	0.20 (0.06-0.33)	.003

a Mean (SD) sum-score is calculated from the 1 to 5 Likert scale where 1 means very low satisfaction and 5 means very high satisfaction.

b Estimates with 95% CIs, analyzed by bootstrapping linear regression.

### Intrapartum Care

Significant differences in care were observed among women in the intervention group during intrapartum care, with an adjusted mean difference of 0.31 between the groups (95% CI 0.17-0.45; *P* < .001). Women in the MLCC group were, among others, more likely to report feeling reassured about the events occurring during labor and delivery (adjusted mean difference of 0.43, 95% CI 0.27-0.59; *P* < .001). They also indicated that the care they received was delivered competently (adjusted mean difference of 0.48, 95% CI 0.31-0.65; *P* < .001) and that they were treated with respect by midwives (adjusted mean difference of 0.46, 95% CI 0.28-0.63; *P* < .0001) when compared to women in the shared care group (see Table 5).

**Table 5.** Participant’s Satisfaction with Intrapartum Care Among Intervention and Control Groups, Sidama Regional State, Ethiopia.

Care assessed	Mean sum score^a^ midwifery-led continuity of care (N = 236 (100%))	Shared care (N = 233 (100%))	Crude mean difference (95% CI)	Adjusted mean^b^ difference (95% CI)	*P*-value
I was consistently involved in making decisions regarding my care during labor and childbirth.	2.76 (0.63)	2.34 (0.93)	0.42 (0.27-0.56)	0.34 (0.19-0.52)	<.001
The midwives/doctors were consistently encouraging and supportive throughout my labor and delivery experience.	2.85 (0.52)	2.42 (0.91)	0.43 (0.29-0.56)	0.37 (0.20-0.54)	<.001
The midwives provided reassurance whenever I needed it during my labor and birth.	2.82 (0.56)	2.42 (0.90)	0.39 (0.26-0.53)	0.43 (0.27-0.59)	<.001
Care during labor and childbirth was delivered in a safe manner.	2.80 (0.59)	2.42 (0.91)	0.38 (0.24-0.51)	0.38 (0.21-0.54)	<.001
Care during labor and delivery was provided in a competent way.	2.80 (0.60)	2.34 (0.94)	0.46 (0.31-0.60)	0.48 (0.31-0.65)	<.001
I was satisfied with the emotional support I received from the midwives and doctors during my labor and birth experience.	2.48 (0.87)	2.22 (0.97)	0.26 (0.09-0.43)	0.26 (0.06-0.46)	<.009
My privacy needs were well respected during labor and birth.	2.79 (0.61)	2.31 (0.94)	0.47 (0.33-0.61)	0.46 (0.28-0.63)	<.001
Overall-satisfaction during intrapartum.	2.78 (0.47)	2.47 (0.78)	0.31 (0.18-0.42)	0.31 (0.17-0.45)	<.001

a Mean (SD) sum-score is calculated from the 1 to 5 Likert scale where 1 means very low satisfaction and 5 means very high satisfaction.

b Estimates with 95% CIs, analyzed by bootstrapping linear regression.

### Postnatal Care

The study found that women who received MLCC during the postnatal period reported significantly higher satisfaction with their care compared to those who received shared care. The mean satisfaction score was 2.76 for the MLCC group and 2.32 for the shared care, with an adjusted mean difference of 0.44 (95% CI 0.29-0.59, *P* < .001). Women in the MLCC group were more likely to report that midwives provided necessary guidance and support better advice for personal health and recovery in the postnatal ward (adjusted mean difference 0.67, 95% CI 0.52-0.83, *P* < .001); Believe that the care provided in hospital after the birth was provided in a safe and competent way (adjusted mean difference 0.30, 95% CI 0.11-0.49, *P* < .001); Express greater satisfaction with the support and guidance given for breastfeeding (adjusted mean difference 0.52, 95% CI 0.35-0.69, *P* < .001) compared to those who received shared care (see Table 6).

**Table 6.** Participant’s Satisfaction with Postnatal Care Among Intervention and Control Groups, Sidama Regional State, Ethiopia.

Care assessed	Mean sum score^a^ midwifery-led continuity of care (N = 236 (100%))	Shared care (N = 233 (100%))	Crude mean difference (95% CI)	Adjusted mean^b^ difference (95% CI)	*P*-value
Active involvement in care decisions baby and myself.	2.86 (0.50)	2.13 (0.99)	0.72 (0.58-0.86)	0.73 (0.57-0.89)	<.001
Support and guidance for breastfeeding.	2.74 (0.65)	221 (0.97)	0.53 (0.38-0.68)	0.52 (0.35-0.69)	<.001
Received necessary guidance and support.	2.89 (0.44)	2.24 (0.96)	0.65 (0.51-0.78)	0.67 (0.52-0.83)	<.001
Guidance and support for baby-for any problem.	2.63 (0.76)	2.19 (0.97)	0.44 (0.28-0.60)	0.49 (0.31-0.68)	<.001
Advice and support for personal health and recovery.	2.66 (0.75)	2.20 (0.97)	0.45 (0.29-0.61)	0.43 (0.25-0.62)	
Midwives/doctors were compassionate and understanding care.	2.65 (0.75)	2.21 (0.97)	0.44 (0.27-0.59)	0.46 (0.27-0.64)	<.001
Midwives/doctors were supportive and reassuring.	2.67 (0.73)	2.17 (0.98)	0.51 (0.35-0.66)	0.55 (0.37-0.73)	<.001
Care in hospital after the birth was provided in a safe and competent way.	2.52 (0.85)	2.26 (0.96)	0.26 (0.09-0.42)	0.30 (0.11-0.49)	<.001
Overall, satisfaction with postnatal care.	2.76 (0.52)	2.32 (0.86)	0.44 (0.31-0.56)	0.44 (0.29-0.59)	<.001

a Mean (SD) sum-score is calculated from the 1 to 5 Likert scale where 1 means very low satisfaction and 5 means very high.

b Estimates with 95% CIs, analyzed by bootstrapping linear regression.

### Overall Assessment of Different Aspect of Care

Based on various aspects of care, the composite variables of satisfaction were analyzed using exploratory factor analysis. The items were grouped into the following categories: information provision and relationships with midwives, women’s assessments of perceived quality of care, and overall satisfaction for each care component. Women in the MLCC group reported higher overall satisfaction levels regarding their pregnancy care compared to those in the shared care, with an adjusted mean difference of 0.28 (95% CI 0.13-0.40; *P* < .001). The assessment of women in the MLCC group highlighted significant satisfaction particularly concerning information provision and their relationships with midwives. This was notably reflected in their evaluations of antenatal care, which showed an adjusted mean difference of 0.20 (95% CI 0.13-0.33; *P* < .001).

**Figure 3.**
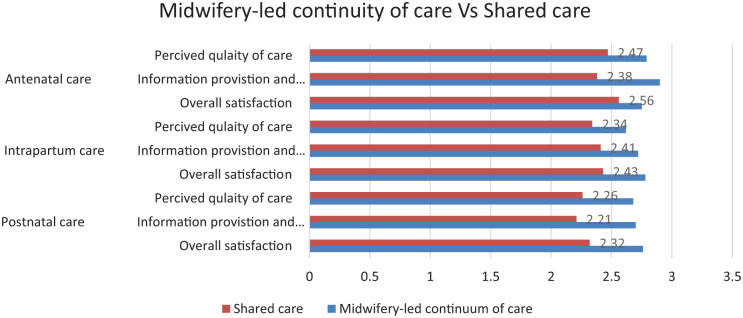
Comparison of women’s evaluations of various aspects of antenatal, intrapartum, and postpartum care, along with an overall assessment of these care experiences, between women in midwifery-led continuity of care (N = 236), and women in shared care (N = 233).

The differences in women’s assessments between the MLCC and shared care groups were most pronounced particularly evident in their overall satisfaction with postnatal care, which had an adjusted mean difference of 0.44 (95% CI 0.29-0.59), see Figure 3.

## Discussion

Women’s satisfaction with maternal health services is a crucial measure of the effectiveness and responsiveness of the healthcare system in meeting their needs and expectations during pregnancy, childbirth and postpartum.^26,27^ This study therefore aimed to investigate and compare the satisfaction levels of women receiving Midwife-Led continuity of care (MLCC) versus those receiving shared care during antenatal, intrapartum, and postpartum care received in general hospitals of the Sidama region in Ethiopia.

This study filled a crucial evidence gap by demonstrating the effectiveness of midwifery-led continuity of care (MLCC) in sub-Saharan Africa. The findings have direct practical implications, supporting maternal health policy reform in Ethiopia to improve care quality and outcomes. Furthermore, it highlights the importance of future research that includes high-risk pregnancies and evaluates both clinical outcomes and patient satisfaction. Such comprehensive evidence is essential to inform policy decisions and successfully scale up MLCC programs, ultimately enhancing maternal and newborn health across the region.

Midwifery-led continuity of care (MLCC) and shared care models offer distinct approaches to maternal healthcare, significantly impacting women’s satisfaction.^18^ The MLCC has been associated with higher satisfaction, as evidenced by studies from different countries.^17,18,21^ In contrast, shared care often involves multiple healthcare providers, resulting in fragmented experiences for patients. This can lead to lower satisfaction levels, as inconsistent support and communication among providers may hinder the continuity of care and create confusion for patients navigating their healthcare journey.^21^ In this study the result showed that, throughout the whole care period, women who received MLCC consistently expressed significantly higher satisfaction with their care compared to shared care, noting that they were well-informed about ongoing processes, received reassurance, participated actively in decision-making during labor, and experienced care that was delivered safely and competently.

In this study, a significant number of women in the intervention group showed a strong preference for seeing the same midwife at every pregnancy check-up. They expressed a desire to have the midwife they became familiar with during their antenatal visits provide care during the intrapartum and postnatal periods. These results are consistent with prior research that underscores the importance of continuity of care.^21,28,29^ Likely, the preference for seeing the same midwife in MLCC stems from a desire for personalized, empowering and supportive care, which ultimately boosts overall satisfaction and enhances maternal health outcomes.^18,30^ However, in contrast to our findings, a literature review conducted by Green et al^31^ found that women who were familiar with their midwife during labor did not report higher satisfaction levels with care compared to those who were not familiar with their midwife. One possible reason is that while familiarity with a midwife can be advantageous, it is not the sole factor affecting satisfaction in MLCC. Overall satisfaction results from a combination of care quality, organizational dynamics, and individual preferences, highlighting the need for a comprehensive approach to care.^32^

Our study reported that women who received MLCC expressed greater satisfaction with antenatal, intrapartum, and postpartum care compared to those who underwent shared care. This satisfaction was notably evident in several important areas, such as emotional support, respect and privacy, safety and competency of the providers, access to information, opportunities for decision-making, and the overall quality of care. Previously conducted studies similarly showed that women involved in MLCC experienced greater levels of satisfaction during antenatal care,^21,28,33-35^ intrapartum,^12,14,21,28,33,35^ and postnatal care.^15,20,21,28,34,35^ Previous evidence also supported that women in MLCC were more likely to be satisfied with their relationships with midwives, the flow of information, the choices and decisions available to them, and the social support they received.^32^ Furthermore, a previous study revealed that the women reported that their privacy was duly respected throughout labor and delivery. Aligning with the findings of the current study, evidence has demonstrated that women in labor highly value the reliability and consistency of midwifery practices.^14,21,35^ This includes attentive and transparent healthcare professionals who provide both physical and emotional support, as those who effectively meet the needs of women during labor are recognized as vital factors influencing their satisfaction with intrapartum care.^9,15,33^ An explanation of the positive findings of MLCC on women’s satisfaction could be that satisfaction among women is the continuity of care provided having dedicated time and attention, emotional support, and fewer medical interventions (cesarean section, episiotomy) contribute to enhanced relationships, access to information, choices, and social support during pregnancy and childbirth. When a pregnant woman knows that the midwife who has cared for her throughout her pregnancy will also be present during labor and provide postnatal care, she experiences a heightened sense of safety and security.^14,36,37^

This study provided essential evidence to support that MLCC could be an alternative approach in maternal and newborn health to improving maternal satisfaction and better utilization of health services. It can inform maternal health policies in the regional and national level in Ethiopia to consider it by highlighting how MLCC can tackle barriers such as fragmented care, low quality, and poor communication skills among providers, which lead to disrespectful treatment and diminished patient satisfaction.^21^ Additionally, it demonstrates how this model empowers women and enhances their experiences during childbirth. Ultimately, this study would contribute to the growing evidence base on MLCC’s benefits in diverse contexts, supporting global efforts to improve maternal health and satisfaction.^7,30^

To implement midwife-led continuity of care (MLCC) in Ethiopia, several essential steps are necessary.^30^ Policymakers must recognize MLCC as a viable alternative approach, ensuring that it aligns with national health objectives to improving maternal and child health, equity in care access, and reducing maternal mortality. This alignment will facilitate the integration of MLCC into the healthcare system and improve maternal and infant health outcomes. Additionally, enhancing infrastructure is essential to provide adequate facilities and resources for midwives and mothers. Furthermore, training programs should be established to build the capacity of healthcare providers in delivering MLCC effectively, and stakeholder engagement is vital to foster collaboration among government bodies, healthcare professionals, and community organizations. Finally, raising awareness among women about the benefits of MLCC can encourage greater utilization of maternal health services, ultimately improving health outcomes in the region.^3^ These elements are vital for promoting and inducing MLCC initiatives. However, as yet MLCC has not been widely adopted in Ethiopia due to challenges such as lack of policy, and inconsistent organizational readiness to embrace new care models.^30^

### Strengths and Limitations

This study used a previously used, validated questionnaire to evaluate satisfaction with MLCC models, ensuring the reliability of its findings.^38,39^ Another strength of the study was that the analytical methods providing a clear overall measure of satisfaction while enhancing validity by controlling demographic imbalances. The other strength of this study was it allows for a direct comparison between MLCC and shared care, which can provide insights into the effectiveness of different maternity care models in terms of patient satisfaction. However, there are limitations to consider. The absence of randomization can introduce selection bias, as women may choose their care model based on personal preferences or circumstances, potentially skewing satisfaction results.

A key limitation of this study was the absence of randomization. This makes it vulnerable to selection bias, where personal preferences in choosing care models can significantly affect outcomes. The lack of randomization also complicates the control of confounding variables, such as socioeconomic status and pre-existing health issues, thereby threatening the accurate assessment of the intervention’s impact. However, we have adjusted the multivariate models in the statistical analyses for potential confounding variables. Nonetheless, potential biases include systematic differences between care groups, and performance bias resulting from variations in care delivery, such as midwives offering more personalized care.

The study exclusively focuses on low-risk women who attended antenatal care (ANC) before 24 weeks of gestation, meaning the results should not be generalized to high-risk pregnancies or women who sought ANC after 24 weeks or did not attend at all, and this may limit the generalizability of the findings to broader maternity care populations. This limitation could lead to disparities in access to essential services and potentially worsen health outcomes for these women. There was also a potential for bias due to the reliance on self-reported data from women, which could lead to overestimations or underestimations of maternal satisfaction levels with MLCC. Additionally,

Since midwives were recruited based on their willingness to participate, this volunteer-based approach may introduce selection bias, potentially overrepresenting positive experiences and excluding a broader range of midwifery competence. This limitation affects the validity of the study’s findings and makes it challenging to draw accurate conclusions about the overall effectiveness, quality, and consistency of the midwifery-led continuity of care (MLCC) model.

## Conclusion

The significance of this study lies in its provision of evidence demonstrating the positive impact of midwife-led continuity of care (MLCC) on maternal satisfaction in a low-resource setting such as Ethiopia. Additionally, this research fills a crucial gap in that the research predominantly comes from high-income countries.^18^

Women in MLCC care reported improved satisfaction compared to women in shared care with the maternal and newborn care in the Sidama region, Ethiopia. Women who received MLCC expressed their satisfaction with their care, noting that they were well-informed about ongoing processes, received reassurance, participated actively in decision-making during labor, and experienced care that was delivered safely and competently, in contrast to those receiving shared care.

This study indicated that this model of care could be an alternative approach to address issues associated with low satisfaction levels by focusing on building strong relationships and by facilitating good communication. However, further research is needed to investigate its effects on high-risk women as well.
